# Investigating lupus retention in care to inform interventions for disparities reduction: an observational cohort study

**DOI:** 10.1186/s13075-020-2123-4

**Published:** 2020-02-22

**Authors:** Christie M. Bartels, Ann Rosenthal, Xing Wang, Umber Ahmad, Ian Chang, Nnenna Ezeh, Shivani Garg, Maria Schletzbaum, Amy Kind

**Affiliations:** 1 Department of Medicine, Rheumatology Division, University of Wisconsin School of Medicine and Public Health, 1485 Highland Ave, Rm 4132, Madison, WI 53705 USA; 2 Health Services & Care Research Program, Department of Medicine, University of Wisconsin School of Medicine and Public Health, Madison, WI USA; 3 Medical College of Wisconsin, Milwaukee, WI USA; 4 Clement J. Zablocki Veterans Affairs Medical Center, Milwaukee, WI USA; 5 Department of Biostatistics, University of Wisconsin School of Medicine and Public Health, Madison, WI USA; 6 Seattle Children’s Hospital, Seattle, WI USA; 7 Department of Medicine, Rheumatology, University of California Irvine Medical Center, Orange County, CA USA; 8 University of Wisconsin School of Medicine and Public Health, Madison, WI USA; 9 Geriatrics Division, Department of Medicine, University of Wisconsin School of Medicine and Public Health, Madison, USA; 10 VA Geriatrics Research Education and Clinical Center, William S Middleton VA Hospital, Madison, WI USA

**Keywords:** Systemic lupus erythematosus, Health disparities, Social determinants of health, Retention in care, Health care quality

## Abstract

**Background:**

Systemic lupus erythematous (SLE) disproportionately impacts patients of color and socioeconomically disadvantaged patients. Similar disparities in HIV were reduced through a World Health Organization-endorsed Care Continuum strategy targeting “retention in care,” defined as having at least two annual visits or viral load lab tests. Using similar definitions, this study aimed to examine predictors of lupus retention in care, to develop an SLE Care Continuum and inform interventions to reduce disparities. We hypothesized that Black patients and those residing in disadvantaged neighborhoods would have lower retention in care.

**Methods:**

Abstractors manually validated 545 potential adult cases with SLE codes in 2013–2014 using 1997 American College of Rheumatology (ACR) or 2012 Systemic Lupus Erythematosus International Collaborating Clinics (SLICC) criteria. We identified 397 SLE patients who met ACR or SLICC criteria for definite lupus, had at least one baseline rheumatology visit, and were alive through 2015. Retention in care was defined as having two ambulatory rheumatology visits or SLE labs (e.g., complement tests) during the outcome year 2015, analogous to HIV retention definitions. Explanatory variables included age, sex, race, ethnicity, smoking status, neighborhood area deprivation index (ADI), number of SLE criteria, and nephritis. We used multivariable logistic regression to test our hypothesis and model predictors of SLE retention in care.

**Results:**

Among 397 SLE patients, 91% were female, 56% White, 39% Black, and 5% Hispanic. Notably, 51% of Black versus 5% of White SLE patients resided in the most disadvantaged ADI neighborhood quartile. Overall, 60% met visit-defined retention and 27% met complement lab-defined retention in 2015. Retention was 59% lower for patients in the most disadvantaged neighborhood quartile (adjusted OR 0.41, CI 0.18, 0.93). No statistical difference was seen based on age, sex, race, or ethnicity. More SLE criteria and non-smoking predicted greater retention.

**Conclusions:**

Disadvantaged neighborhood residence was the strongest factor predicting poor SLE retention in care. Future interventions could geo-target disadvantaged neighborhoods and design retention programs with vulnerable populations to improve retention in care and reduce SLE outcome disparities.

## Background

Systemic lupus erythematosus (SLE) is an autoimmune disease that disproportionately impacts young women, patients of color, and the socioeconomically disadvantaged, making SLE an important target for health disparity measurement and research [1]. Despite many effective lupus monitoring strategies and treatments [2, 3], significant healthcare disparities and outcome gaps remain [4–6]. Compared to White women, the US Black women are up to seven times more likely to develop renal failure, and two to three times as likely to die prematurely [4, 7]. SLE is also a leading chronic disease cause of death in the US women ages 18–25 [8]. Arguing against a purely biological mechanism, higher SLE damage was noted in the US African descendants but not in other African descendants in ten other countries in the SLICC cohort [9]. High-quality clinical care can reduce risk for many poor lupus outcomes [10, 11]. Yet, some data suggest that Black or low SES patients with milder onset SLE are more likely to ultimately die of their disease, suggesting that differences in healthcare follow-up might be to blame [7, 12].

**Fig. 1:**
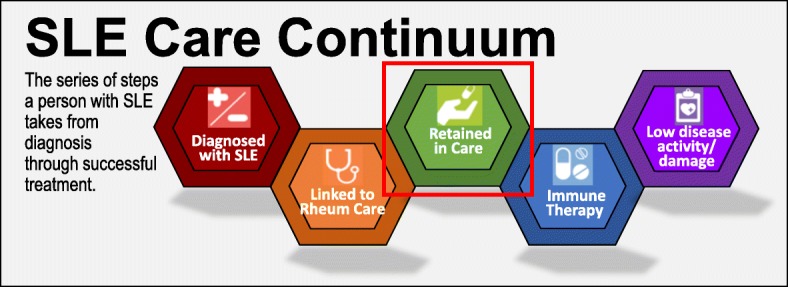
Proposed SLE Care Continuum adapted with permission from the HIV.gov Care Continuum

Human immunodeficiency virus (HIV), which also disproportionately impacts young, Black, and socioeconomically disadvantaged populations, is an analogous condition where healthcare gaps have been targeted as a means by which to reduce disparities. As a framework, experts at the World Health Organization (WHO) [13], US Institute of Medicine (IOM) [14], and Centers for Disease Control and Prevention (CDC) [15] have defined an “HIV Care Continuum” outlining five critical healthcare steps to achieve successful viral suppression. The Care Continuum offers standard definitions to measure gaps at each step from (1) diagnosis to (2) linkage with specialty HIV care, (3) retention in care, and (4) retention on antiretroviral therapy, and finally to (5) control with suppressed HIV viral loads [16]. Using this continuum, HIV researchers determined that gaps in retention in care account for most (61%) failures to control HIV [16]. Moreover, continuum research has led to a compendium of evidence-based interventions [15] to close gaps at each step including many interventions to improve retention in HIV care. A similar approach holds promise for improving disparities in SLE. As such, we proposed an SLE Care Continuum from (1) diagnosis to (2) linkage with specialty lupus care, (3) retention in care, (4) retention on immune therapy, and finally (5) low lupus disease activity (Fig. 1). In this study, we first focus on predictors of retention in care.

Rheumatologists often observe that keeping some lupus patients, particularly young, Black, or socioeconomically disadvantaged lupus patients [17], in follow-up care is a challenge, yet no metrics exist to identify who is or is not retained in lupus care. In HIV, retention in care is defined by WHO [13] and CDC [15] experts as having two annual HIV visits or one viral load every 6 months. Creating such metrics was pivotal for identifying gaps and testing strategies to reduce HIV health disparities [15]. Despite an urgent need to reduce SLE disparities and known concerns regarding retention, we have no established methods to measure gaps in retention in care or other disparities across an SLE Care Continuum.

We were particularly interested in investigating relationships between race, socioeconomic disadvantage, and retention in lupus care in this cohort [18]. Black patients and the socioeconomically disadvantaged are more likely to reside in disadvantaged US neighborhoods, which are linked to high disease rates and mortality offering geographic targets for policy, practice change, and research [19, 20].

We hypothesized that Black patients and those residing in highly disadvantaged neighborhoods would have lower retention in care because of the multitude of socioeconomic, access, and other adverse social determinants of health that such residents face [21–23]. The objectives of this study were to validate an urban lupus cohort, to create metrics for retention in care, and to examine how race and other social determinants of health predict lupus retention in care. Defining lupus retention in care and its predictors are key steps toward designing future interventions to improve retention and reduce lupus outcome disparities.

## Methods

### Inclusion and study population

For this cohort study, we first searched all Jan 2013–June 2014 electronic health records (EHR) at an urban US academic center for all inpatient or outpatient visits with the International Classification of Diseases ninth or tenth edition (ICD-9 or 10) codes representing possible SLE [ICD-9 710.0 or ICD-10 M32, M32.1, M32.8, M32.9]. Inclusion required being age 18 or over, living through 2015 to assure eligibility for retention in care, and having at least one ambulatory visit in rheumatology [physician (MD, DO), nurse practitioner (NP), physician assistant (PA), resident, or fellow] and one in primary care (family medicine, internal medicine, geriatrics, obstetrics and gynecology, or pediatrics) in 2013–2014 to assure equal capture of baseline comorbidity and healthcare utilization information and follow-up. Recognizing that not all patients with lupus diagnosis codes meet clinical criteria, trained health professionals manually validated all potential SLE cases using a standard REDCap abstraction tool to assess the 1997-modified 1982 American College of Rheumatology (ACR) classification criteria and 2012 Systemic Lupus Collaborating Clinics (SLICC) SLE classification criteria [24]. Laboratory, pathology, imaging, and clinical note data were reviewed for all patients with possible lupus. Those with drug-induced lupus or not meeting either definite classification of SLE were excluded. All patients meeting either the 1997 ACR or 2012 SLICC SLE classification criteria and other inclusion criteria were included in the study. Patients were followed from the study start date of their first lupus diagnosis code, through death, or the end of 2015 as a study end date. Data elements for each individual were linked via a unique pseudo identifier, and direct identifiers were removed for a final limited dataset for analysis.

The Institutional Review Board approved of this minimal risk medical record review study with a waiver of individual informed consent and use of a limited dataset.

### Data sources

In addition to manually abstracted items, EHR data were electronically extracted for definite SLE cases including (1) patient-level sociodemographic variables (age, sex, race, ethnicity, rural-urban commuting area (RUCA) classification, date of death), (2) visit-level information (all rheumatology encounter dates and all encounter ICD codes), and (3) select laboratory data (e.g., C3/C4 complement and anti-double-stranded DNA antibody testing dates). In addition to SLE classification criteria, sociodemographic and behavioral factors also reviewed during manual abstraction were nine-digit ZIP postal code and smoking history. Disease severity was noted using the number of SLE criteria, and a history of lupus nephritis was also manually validated.

### Outcome definitions for lupus retention in care

EHR dates of rheumatology visits attended and complete blood count (CBC), creatinine anti-double-stranded DNA (dsDNA), or C3 or C4 complement tests during the calendar year 2015 were used to test potential retention definitions. Thresholds of one, two, or four visits or complement or dsDNA lab tests were compared. We focused on two visit-defined and two lab-defined SLE retention in care in those without nephritis analogous to HIV definitions and consistent with guidelines for nephritis screening and quality measures [2, 11]. We also tested a four lab-defined threshold consistent with SLE guidelines for those with lupus nephritis [2, 3]. Non-specific tests such as complete blood counts and creatinine were tested but not used as final definitions as they may be ordered by other providers for other non-SLE reasons and may not indicate lupus retention in care. Tests such as urinalyses, while important to most lupus care, were avoided given that SLE patients on dialysis may forgo such testing or have results at external dialysis centers and thus not present in EHR data.

### Predictors and covariates

Consistent with the National Institute of Minority Health and Health Disparities research framework [21], multilevel predictors of interest included individual-level age, sex, race, ethnicity, and smoking status, and ZIP code linked contextual-level rural urban (RUCA) classification, and neighborhood disadvantage quartile. We used the Area Deprivation Index (ADI), a neighborhood disadvantage metric encompassing 17 education, employment, housing quality, transportation, and poverty measures to match from 69 million nine-digit ZIP postal code census block groups, i.e., “neighborhoods” of approximately 1500 people [19, 20, 25]. Twenty-five patients were missing residential ADI data and were excluded from multivariable modeling. The number of ACR criteria and any history of nephritis were included as markers of lupus severity.

### Statistical analysis

Baseline lupus cohort characteristics were presented as frequencies and proportions for the overall and the cohort stratified by race. Differences between proportions were examined by the chi-square test. Retention rates during the 2015 period were calculated as percent of eligible patients meeting visit or laboratory testing definitions. We also examined definitions specific to guideline-recommended lab frequencies for those with lupus nephritis [2, 12]. Next, to determine predictors of retention, univariate and multivariable logistic regression analyses were performed, and results are presented as odds ratios (ORs) along with 95% confidence intervals (CIs). Potential interactions between ADI neighborhood disadvantage and other covariates (e.g., race or ethnicity) were tested as sensitivity analyses. We a priori estimated 80% power to detect an odds ratio of 0.60 if retention in care is 65% in White compared to 53% in Black patients using a two-sided *Z* test, significance level 0.05, similar to a 15% reported race gap in medication adherence [26]. Analyses were completed using SAS version 9.4 (Cary, NC) and STATA version 13.1 (College Station, TX).

## Results

### Cohort description

**Fig. 2:**
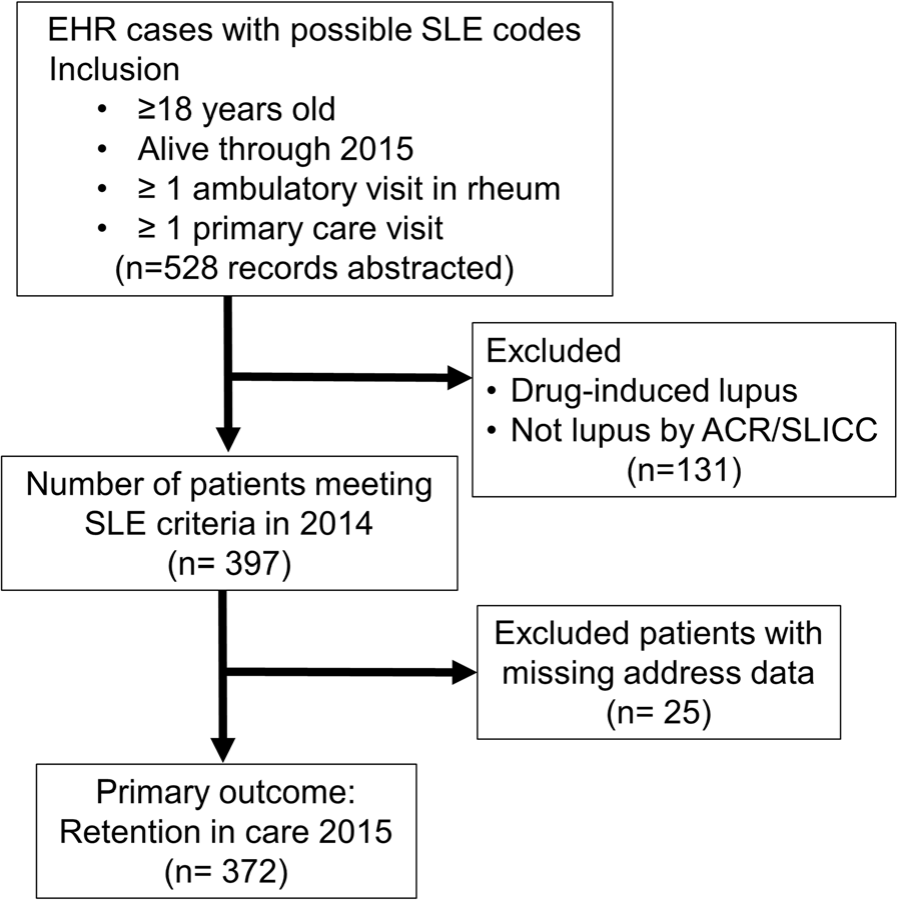
Flow diagram of project design and inclusion

Of 528 abstracted potential lupus cases, 397 patients met ACR or SLICC classification criteria for definite lupus and were included in our study (Fig. 2). Among definite lupus patients, 91% were female, 39% Black, 5% Other, and 4% Hispanic (see Table 1).

**Table 1:** Prevalent systemic lupus erythematosus cohort description (*n* = 397)

	Total cohort	White/other	Black	*p*
	*n* = 397	*n* = 241	*n* = 156	
Age category				
18–29	54 (13.6%)	30 (12.5%)	24 (15.4%)	< 0.001
30–40	96 (24.2%)	50 (20.8%)	46 (29.5%)	
40–60	175 (44.1%)	102 (42.3%)	73 (46.8%)	
60–80	67 (16.9%)	56 (23.2%)	11 (7.1%)	
80+	5 (1.3%)	3 (1.2%)	2 (1.3%)	
Female	361 (90.9%)	219 (90.9%)	142 (91.0%)	0.96
Race				
White	221 (55.7%)	221 (91.7%)	NA	< 0.001
Black	156 (39.3%)	NA	156 (100%)	
Other	20 (5.0%)	20 (8.3%)	NA	
Ethnicity				
Hispanic	17 (4.3%)	16 (6.6%)	2 (1.3%)	0.01
RUCA				
Urban	329 (82.9%)	175 (72.6%)	154 (98.7%)	< 0.001
Suburban	36 (9.1%)	36 (14.9%)	0 (0%)	
Large town	15 (3.8%)	14 (5.8%)	1 (0.6%)	
Small town	17 (4.3%)	16 (6.6%)	1 (0.6%)	
Smoking (ever)	168 (42.3%)	103 (43%)	65 (42%)	0.60
Payer				
Commercial	176 (44.3%)	139 (57.7%)	37 (23.7%)	< 0.001
Medicaid	71 (17.9%)	24 (10.0%)	47 (30.1%)	
Medicare	142 (35.8%)	72 (29.9%)	70 (44.9%)	
Uninsured/unknown	8 (2.0%)	6 (2.5%)	2 (0.06%)	
Neighborhood disadvantage by ADI quartile				
1st (least disadvantage)	93 (25.0%)	83 (34.4%)	10 (6.4%)	< 0.001
2nd	93 (25.0%)	78 (32.4%)	15 (9.6%)	
3rd	93 (25.0%)	54 (22.4%)	39 (25.0%)	
4th (most disadvantage)	93 (25.0%)	13 (5.4%)	80 (51.3%)	
Positive ACR criteria	365 (91.9%)	210 (87.1%)	148 (94.9%)	0.02

Compared to White patients with lupus, Black patients were younger, more likely to reside in urban areas, and 10 times more likely to reside in a neighborhood within the most disadvantaged quartile (51% Blacks vs. 5% Whites). Black patients were also more likely to meet ACR SLE criteria (95% vs. 87%). Payer varied significantly by race (75% of Black versus 40% of White patients received public insurance), and only 2% of patients were uninsured or had unknown payer status.

### Testing retention definitions

When examining definitions of lupus retention in care, we first examined visit-based definitions similar to those employed in HIV. Overall, as shown in Table 2, 83% (*n* = 331) of eligible lupus patients had at least one ambulatory rheumatology visit in 2015, and 60% (*n* = 238) had at least two visits. This was comparable to 74% with two or more visits in the baseline year 2014 (data not shown).

**Table 2:** Examining visit- and lab-defined retention in care thresholds in SLE cases with and without nephritis (*n* = 397)

		All SLE		Lupus nephritis		*p*
		*n* = 397		*n* = 145		
Definition	Interval	*n*	%	*n*	%	
Visit definitions						
Rh visits	≥ 1/year	331	83	120	83	0.802
*Rh visits	≥ 2/year	238	60	96	66	0.054
Rh visits	≥ 4/year	79	20	46	32	< 0.001
Lab definitions						
C3/C4	≥ 1/year	184	46	78	54	0.024
*C3/C4	≥ 2/year	106	27	49	34	0.015
C3/C4	≥ 4/year	30	8	20	14	< 0.001
CBC	≥ 1/year	213	54	90	62	0.011
CBC	≥ 2/year	159	40	72	50	0.001
CBC	≥ 4/year	93	23	52	36	0.003
Creatinine	≥ 1/year	211	53	89	61	0.013
Creatinine	≥ 2/year	163	41	74	51	0.002
Creatinine	≥ 4/year	92	23	49	34	< 0.001
dsDNA	≥ 1/year	149	38	67	46	0.007
dsDNA	≥ 2/year	77	19	39	27	0.004
dsDNA	≥ 4/year	18	5	12	8	0.007

*Abbreviations*: *Rh* rheumatology MD, DO, NP, PA, or fellow; *C3/C4* complement component 3 or 4 lab tests which were each independently assessed yielding identical results that are shown together; *CBC* complete blood counts; *dsDNA* double-stranded DNA antibody test

*Final models used two visits and at least two labs per year consistent with WHO/CDC HIV definitions and ACR SLE guidelines

When testing lab-defined retention in care, we sequentially examined C3 and C4 complement, CBC, creatinine, and anti-dsDNA testing (Table 2). In 2015 follow-up, 54% of patients had at least one CBC and 46% of patients had at least one complement result recorded in the EHR. Only 27% and 41% had two annual complement and creatinine tests, respectively, compared to 43% with at least two complement tests in the 2014 baseline year (data not shown). Any single anti-dsDNA result was available on only 29% of the cohort during 2015, with only 19% having two dsDNA tests, leading us to operationalize the equally specific two complement test definition for lab-defined lupus retention in care for multivariable modeling although other definitions could be assessed in future studies.

### Predictors of lupus retention in care

In adjusted multivariable models, residing in the neighborhood quartile with worst disadvantage was the strongest predictor of lower retention in care, predicting nearly 60% lower odds (OR 0.41, CI 0.18, 0.93; Table 3). Smoking likewise significantly predicted lower visit-defined retention in care (OR 0.63, 0.40, 0.99). Neither Black race nor Hispanic ethnicity was predictive of lupus retention in care.

**Table 3:** Predictors of two visit-defined lupus retention in care (*n* = 372, multivariable analysis included 372 of 397 SLE patients with complete data)

	Unadjusted OR (95% CI)	Adjusted OR (95% Cl)
Age		
18–29	Ref	Ref
30–40	0.95 (0.47, 1.93)	0.81 (0.36, 1.81)
40–60	0.65 (0.34, 1.24)	0.65 (0.31, 1.36)
60–80	0.62 (0.29, 1.30)	0.71 (0.30, 1.68)
80+	0.75 (0.11, 4.90)	1.15 (0.11, 12.38)
Female	0.73 (0.35, 1.50)	0.50 (0.22, 1.12)
Race		
White	Ref	Ref
Black	1.24 (0.81, 1.89)	1.56 (0.81, 3.03)
Other	0.71 (0.29, 1.78)	0.98 (0.29, 3.33)
Ethnicity (Hispanic)	0.52 (0.20, 1.34)	0.63 (0.18, 2.28)
RUCA		
Urban	Ref	Ref
Suburban	1.03 (0.51, 2.08)	1.19 (0.54, 2.65)
Large town	1.31 (0.44, 3.91)	1.12 (0.33, 3.88)
Small town	0.46 (0.17, 1.23)	0.41 (0.14, 1.23)
Ever Smoking	**0.63 (0.42, 0.95)**	**0.63 (0.40, 0.99)**
Payer		
Commercial	Ref	
Medicaid	0.72 (0.41, 1.25)	
Medicare	1.25 (0.79, 1.98)	
Neighborhood disadvantage by ADI		
1st (least disadvantage)	Ref	Ref
2nd quartile	1.05 (0.58, 1.88)	1.02 (0.55 (1.92)
3rd quartile	1.26 (0.69, 2.29)	1.01 (0.52, 2.00)
4th (most disadvantage)	0.74 (0.41, 1.32)	**0.41 (0.18, 0.93)**
Number of ACR criteria	**1.13 (1.01, 1.27)**	1.10 (0.95, 1.27)
Lupus nephritis	1.52 (0.99, 2.32)	1.35 (0.78, 2.36)

Examining unadjusted odds using the two complement definitions of retention, nephritis predicted greater retention (OR 1.70, CI 1.12, 2.59; Table 4); small town residence predicted lower lab-defined retention. However, in multivariate models, nephritis patients did not have significantly different retention (OR 1.41, CI 0.82, 2.41) and only a higher number of SLE criteria predicted greater retention (OR 1.17, CI 1.01, 1.35).

**Table 4:** Predictors of two complement lab-defined retention in care (*n* = 372, multivariable analysis included 372 of 397 SLE patients with complete data)

	Unadjusted OR (95% CI)	Adjusted OR (95% CI)
Age		
18–29	Ref	Ref
30–40	0.93 (0.47, 1.86)	1.10 (0.51, 2.39)
40–60	1.28 (0.68, 2.39)	1.68 (0.83, 3.41)
60–80	0.89 (0.42, 1.88)	1.40 (0.59, 3.30)
80+	1.13 (0.17, 7.37)	2.94 (0.36, 24.28)
Female	0.77 (0.39, 1.54)	0.77 (0.36, 1.64)
Race		
White	Ref	Ref
Black	1.29 (0.85, 1.97)	0.82 (0.43, 1.55)
Other	1.18 (0.46, 3.00)	1.15 (0.34, 3.92)
Ethnicity (Hispanic)	1.00 (0.38, 2.65)	0.99 (0.28, 3.61)
RUCA		
Urban	Ref	Ref
Suburban	0.90 (0.44, 1.84)	1.32 (0.59, 2.95)
Large town	1.82 (0.64, 5.13)	1.63 (0.49, 5.43)
Small town	**0.87 (0.50, 0.78)**	0.95 (0.32, 2.86)
Ever Smoking	1.08 (0.72, 1.63)	1.01 (0.64, 1.59)
Payer		
Commercial	Ref	
Medicaid	0.92 (0.52, 1.62)	
Medicare	1.13 (0.72, 1.77)	
Neighborhood disadvantage by ADI		
1st (least disadvantage)	Ref	Ref
2nd quartile	0.87 (0.47, 1.59)	0.80 (0.42, 1.51)
3rd quartile	1.20 (0.66, 2.18)	1.10 (0.56, 2.15)
4th (most disadvantage)	1.56 (0.88, 2.82)	1.39 (0.94, 3.04)
Number of ACR criteria	1.19 (1.06, 1.33)	**1.17 (1.01, 1.35)**
Lupus nephritis	**1.70 (1.12, 2.59)**	1.41 (0.82, 2.41)

Among 145 patients with lupus nephritis, slightly more (66 vs. 60%) met the two visit-defined retention criteria. Likewise, 34% vs. 27% had at least two complement tests, but in such patients, only 14% had four tests per year as recommended per ACR guidelines for lupus nephritis [2].

## Discussion

Using definitions analogous to those used by the WHO, IOM, and CDC [13–15] to define HIV retention in care, we were able to define and examine predictors of lupus retention in care. We examined both visit and lab-defined retention in care noting that 40% of patients lacked recommended visits and more than half of patients lacked recommended lupus labs. Notably, our visit-based models revealed that neighborhood disadvantage was the strongest predictor of gaps in lupus retention in care. Patients with lupus nephritis performed slightly better using basic measures, but only 14% met guideline recommended quarterly lab-defined retention.

More broadly, our data, and those of others [27, 28], support defining and investigating a Care Continuum across lupus care to inform the design of targeted interventions to eliminate disparities. Supporting the idea that the SLE Care Continuum would likewise correlate with outcomes, prior studies have shown retention on therapy for instance links to patient outcomes in SLE. Specifically, hydroxychloroquine improves outcomes [6], and nonadherence gaps correlate with worse outcomes, particularly, in disparities populations [27, 28]. A Canadian study correlated better hydroxychloroquine adherence with 5-year reductions in cumulative steroid use, disease activity, and lupus damage [28]. Conversely, a US Medicaid study reported that hydroxychloroquine therapy gaps (as defined by < 80% medication possession) were seen in 83% of patients and correlated with increased emergency visits and hospitalizations by 4 months [27]. We focused upstream in the Care Continuum noting that retention in care is critical to receive prescriptions for such treatments, to decrease treatment interruptions, and to signal treatment escalation when necessary.

Based on prior literature describing outcome disparities and follow-up gaps in Black patients or those from socioeconomic disadvantaged backgrounds [17], we predicted lower retention in care in those groups. We observed that neighborhood disadvantage was most strongly predictive, independent of race. Geographically, our site for this study, urban Milwaukee, WI, USA, may be more segregated than other cities [29] influencing the relative weights of predictors. We observed that 51% of Black patients with SLE resided in the most disadvantaged quartile neighborhoods compared to only 5% of White patients. Both race and neighborhood disadvantage in addition to other factors may be predictive of retention in care in other places, yet data suggest that neighborhood disadvantage predicts health independent of individual socioeconomic status as well [30]. Neighborhood context can directly affect access to healthcare as well as access to transportation, food, education, health behaviors, safety, discrimination, and chronic stress influencing many health outcomes [22, 23, 31]. Mechanistically, if transportation is less available or healthcare facilities are farther from disadvantaged populations, as shown in a US SLE Medicaid study [32], then getting routine visits and lab tests is more challenging. A strength of neighborhood disadvantage as a disparities predictor is that it can be calculated using already collected addresses within EHRs to identify patients who may need additional resources. It can also be used as a first step toward additional targeted research to disentangle the myriad of mechanisms that may underlie lupus disparities and to specifically target these actionable factors for interventions across policy, research, and clinical domains.

Retention in care is a well-developed metric in the HIV literature that has been central to the WHO and US National HIV/AIDS Strategy [15] and for measuring progress in reducing disparities [33]. Among those retained in care, 89% received treatment and 80% achieved viral suppression control, highlighting that retention in care is a key target. Encouragingly, a meta-analysis [34] and the compendium [15] highlight numerous evidence-based interventions proven to improve retention in HIV care and reduce disparities. Policy-driven programs have used such HIV Care Continuum strategies to increase viral suppression from 69 to 81% among Black participants between 2012 and 2016 [35]. Individual patients with HIV now also know that two viral load lab tests per year define a minimum standard to share accountability. Overall, success reducing disparities in HIV highlights the need to measure SLE retention in care as a necessary first step toward designing interventions, policies, and building patient partnerships to eliminate lupus outcome disparities.

Despite strengths of carefully validating lupus cases, applying a novel Care Continuum from HIV, and testing associations using multivariable regression, we also acknowledge limitations. First, our analysis included only one health system in one US city. Future projects will test retention definitions in other health systems or regions. Second, patients with nephritis or skin predominant SLE may be followed more closely in nephrology or dermatology which was not examined in this study. Future studies could examine retention in care including visits provided by nephrologists or dermatologists, who may share care for patients with SLE. A strength of our EHR-derived visit-based approach to define retention is the possibility of reproducing this metric across or between health systems. It is possible that compared to single-system EHR assessments, payer claims datasets might show higher retention in care given capture of visit or lab claims anywhere, including labs performed in other local health systems. For instance, we noted that small town residents had lower univariate odds of complement tests; some may have had lab testing in local clinics while others simply did not have tests. Likewise, only 2% of our cohort was uninsured and retention rates may vary in populations with more patients who are uninsured. We also acknowledge that some patients may have been lost to follow-up—migrating or intentionally changing insurance or providers, without true gaps in lupus retention in care. Residence may also change more frequently in patients with greatest disadvantage or adverse life experiences [36, 37]. Such time-varying factors could be examined in future multi-site studies.

## Conclusions

Defining lupus retention in care and its predictors are key steps toward future evidence-based interventions to improve retention and reduce some disparities in lupus outcomes. Residence in a disadvantaged neighborhood was the strongest factor predicting poor SLE retention in care. Constructing future interventions could leverage geo-targeting disadvantaged neighborhoods, as well as designing programs specifically with and for vulnerable populations residing within these neighborhoods. Ultimately, we aim to use the SLE Care Continuum measures to design evidence-based care strategies and policies to reduce health disparities and advance outcome equity among patients with lupus.

## Data Availability

The datasets generated and analyzed during the current study are not publicly available due to limited patient health information but may be reviewed with the corresponding author on reasonable request.

## Abbreviations

ACR: American College of Rheumatology
ADI: Neighborhood area deprivation index
CDC: Centers for Disease Control and Prevention
*DNA*: Deoxyribonucleic acid
*dsDNA*: *Double stranded DNA antibody test*
EHR: Electronic health record
HIV: Human immunodeficiency virus
ICD: International Classification of Diseases
IOM: US Institute of Medicine
RUCA: Rural-urban commuting area
SLE: Systemic lupus erythematosus
SLICC: Systemic Lupus Erythematosus International Collaborating Clinics
WHO: World Health Organization
